# S100A8-Mediated Inflammatory Signaling Drives Colorectal Cancer Progression via the CXCL5/CXCR2 Axis

**DOI:** 10.7150/jca.92588

**Published:** 2024-04-29

**Authors:** Libin Chen, Peng Shu, Xuemei Zhang, Shuyu Ye, Li Tian, Shourong Shen, Jian Ma, Feiyan Ai, Xiayu Li

**Affiliations:** 1 Department of Gastroenterology, The Third Xiangya Hospital of Central South University, Changsha, Hunan, China.; 2 Hunan Key Laboratory of Nonresolving Inflammation and Cancer, Central South University, Changsha, Hunan, China.; 3 Department of Gastroenterology, Anhui No.2 Provincial People's Hospital.; 4 Department of Pathology, Liuzhou People's Hospital Affiliated to Guangxi Medical University, No. 8 Wenchang Road, Liuzhou 545006, China.; 5 Hunan Key Laboratory of Cancer Metabolism, Hunan Cancer Hospital and the Affiliated Cancer Hospital of Xiangya School of Medicine, Central South University, Changsha, China.; 6 Key Laboratory of Carcinogenesis and Cancer Invasion of the Chinese Ministry of Education, NHC Key Laboratory of Carcinogenesis, Cancer Research Institute, School of Basic Medical Science, Central South University, Changsha, China.

**Keywords:** S100A8/S100A9, colorectal cancer, inflammatory cytokine, signaling pathways, NF-κB, ERK-MAPK, CXCL5/CXCR2

## Abstract

**Background:** S100A8/S100A9 belong to the S100 calcium-binding protein family and play an essential role in the progression of chronic inflammation in diseases. It also regulates various biological processes such as tumor cell survival, apoptosis, and invasive metastasis. The extracellular form of S100A8/S100A9 functions by modulating cellular oxidative metabolism and facilitating inflammation-to-cancer progression. This modulation occurs through specific binding to receptors like RAGE and TLR4 and activation of signaling pathways including STAT3 and NF-κB. In tumor cells, S100A8 and S100A9 induce phenotypic changes by influencing calcium ion concentrations and other pathways. However, the precise function of high S100A8/S100A9 expression in colorectal cancer cells remains unclear.

**Methods:** To explore the role of S100A8/S100A9 in colorectal cancer, we used immunohistochemistry and data from GEO and TCGA databases to analyze its expression in colorectal cancer cells, normal intestinal mucosa, and adjacent tissues. Functional models of high S100A8/S100A9 expression in colorectal cancer cells were established through transfection with overexpression plasmids. Protein microarrays, enzyme-linked immunosorbent assays (ELISAs), and real-time PCR were employed to assess the expression and secretion of 40 cytokines. MTT and Transwell invasion assays were conducted to evaluate changes in cell proliferation, invasion, and chemotaxis. Finally, tail vein and subcutaneous tumorigenesis assays assessed cell proliferation and migration in vivo.

**Results:** We observed significantly higher expression of S100A8/S100A9 in colorectal cancer epithelial cells compared to normal intestinal mucosa and adjacent tissues. Overexpression of S100A8/S100A9 in mouse colon cancer cells CT26.WT led to differential increases in the secretion levels of various cytokines (CXCL5, CXCL11, GM-CSF, G-CSF, IL1a, IL1b, sTNF RI, and CCL3). Additionally, this overexpression activated signaling pathways such as STAT3, NF-κB, and ERK-MAPK. The synthesis and secretion of inflammatory factors could be inhibited by using NF-κB and ERK-MAPK pathway inhibitors. Moreover, S100A8 promotes the proliferation and invasion of colon cancer cells. Notably, the CXCR2 inhibitor (SB265610) effectively reversed the phenotypic changes induced by the CXCL5/CXCR2 biological axis.

**Conclusions:** Our findings indicate that increased expression of S100A8 and S100A9 in colon cancer epithelial cells enhances the secretion of inflammatory factors by activating NF-κB, ERK-MAPK, and other signaling pathways. S100A8 facilitates colon cancer cell proliferation, invasion, and metastasis through the CXCL5/CXCR2 biological axis.

## Introduction

Colorectal cancer (CRC) is the third leading cause of cancer-related fatalities worldwide, with over 1.85 million cases and approximately 850,000 annual deaths 1. In China, the year 2020 saw about 555,000 new cases of colorectal cancer, alongside rising incidence and mortality rates 2. Among the key risk factors, inflammatory bowel disease (IBD) ranks as one of the top three predisposing factors for colorectal cancer. Although IBD-associated colorectal cancer (IBD-CRC) accounts for only 1-2% of all colorectal cancer cases, individuals with IBD colitis have a six-fold higher susceptibility to colorectal cancer than the general population 3, 4. Notably, colorectal cancer contributes to 10-15% of all deaths among patients with IBD, marked by an increased prevalence of multiple synchronous colorectal cancers 5. Research has shown that the genesis of inflammatory bowel disease-associated colorectal cancer (CAC) arises from sustained activation of inflammation-related signaling pathways like NF-κB, coupled with a persistent inflammatory microenvironment, ultimately leading to the development of colorectal epithelial cell carcinogenesis 6, 7.

We established a dynamic mouse model to explore the relationship between colonic inflammation and cancer in mice treated with DSS/AOM 8. Throughout all stages, from inflammation to tumor development, we noted a significant increase in the expression of S100A8/A9. S100A8/A9 belongs to the S100 protein family and is characterized by two EF-hand calcium-binding domains 9, 10. These domains are essential for calcium ion regulation and form the structural basis for the functionality of the protein family 11. Moreover, S100A8 and S100A9 serve as alarm signals for inflammation and are involved in oncogenic processes 12, 13. Under pathological conditions such as mechanical injury, infection, and immune disorders, bone marrow-derived cells—including neutrophils, monocytes, fibroblasts, tumor-associated phagocytes, and bone marrow-derived suppressor cells 14-17—synthesize and release substantial quantities of S100A8 and S100A9. These proteins act in both autocrine and paracrine manners, entering the cellular interstitial space to modulate the migration and adhesion of inflammatory cells, directing neutrophils toward the site of injury, enhancing inflammatory responses, combating infections, and facilitating tissue repair. Extracellular secretion of S100A8/A9 can directly interact with cell surface receptors such as RAGE and TLR4^18^, ^19^, activating signaling pathways like NF-κB and MAPK 20, 21. This leads to increased secretion of inflammatory factors, causing uncontrolled local inflammation, cellular dysfunction, initiation of malignant transformations in epithelial cells, promotion of tumor cell proliferation, invasion, metastasis, and an overall increase in malignancy, substantially affecting prognosis 22.

Studies have consistently reported elevated S100A8/A9 expression in both inflammatory bowel disease and colorectal cancer tissues 21, 23-25. Additionally, increased levels of S100A8/A9 have been found in various body regions, including the lungs and liver of colorectal cancer patients, contributing to the formation of pre-metastatic niches and affecting distant tumor metastasis 21, 26. Notably, under neoplastic conditions, S100A8 and S100A9 are not solely derived from mesenchymal cells but are also highly expressed in tumor epithelial cells 27. They are secreted into the extracellular matrix and remain intracellular, primarily serving to promote tumor cell deterioration. This occurs through the regulation of intracellular calcium ion concentrations, modulation of key enzyme phosphorylation, and interactions with cytoskeletal proteins 28. However, it is worth noting that there is still a limited body of research on the role of S100A8/A9 in colorectal cancer cells and their associated mechanisms.

The aim of this study was to elucidate the effects and potential mechanisms of high S100A8 and S100A9 expression in colon cancer cells on the secretion of inflammatory factors and malignant transformation of tumors. Our findings demonstrate that overexpression of S100A8 and S100A9 promotes the synthesis and secretion of various inflammatory factors and chemokines through the activation of NF-κB, ERK-MAPK, and other signaling pathways. Moreover, S100A8 enhances the proliferation and invasion of colon cancer cells, mediated by the chemokine CXCL5 and its receptor CXCR2.

## Materials and Methods

### Tissue Specimens

We acquired frozen tissue specimens from 17 individuals diagnosed with colorectal cancer and their corresponding neighboring control tissues at the Third Xiangya Hospital of Central South University. The control tissue samples were extracted from colorectal tissues situated 5 cm away from the tumor site. The selected patients had no prior history of radiotherapy, were not afflicted by significant tobacco or alcohol addiction, had no familial tumor history, and were free from serious chronic conditions like diabetes mellitus, liver, kidney, or hematological disorders. All patients received a clear diagnosis through clinicopathology and were staged using the UICC/AJCC TNM staging system for colorectal cancer. Thorough histological reviews of all tissue sections were conducted by two pathologists to ensure the precision of patient selection. Additionally, the study was formally approved by the Ethics Review Committee of Xiangya Third Hospital of Central South University.

### Cell Culture

Mouse colon cancer cells (CT26.WT), RAW264.7 macrophages, and human colon cancer cells (SW620, SW480.WT) were procured from the Shanghai Cell Bank of the Chinese Academy of Sciences. These cell lines were cultured in RPMI 1640 or DMEM-High Sugar Medium supplemented with 10% imported fetal bovine serum (FBS). Subsequently, the cells were incubated in a bio-incubator under controlled conditions at a temperature of 37°C with a CO2 concentration of 5%.

### Plasmids and Reagents

To achieve overexpression, we designed plasmids carrying full-length cDNAs of S100A8 and S100A9 from mouse and human sources, respectively. Through DNA sequencing, we ensured that the plasmids, namely pIRES-mS100A8, pIRES-mS100A9, pEGFP-hS100A8, and pEGFP-hS100A8, precisely matched the base sequences of their respective cDNAs. The following inhibitors were utilized in the experiments: pD098059 (ERK inhibitor, #S1805) was purchased from Kangwei Century; BAY11-7082 (NF-κB inhibitor, #S1523) was purchased from Byotime; AG490 (STAT3 inhibitor, #S1143) was purchased from Selleck; SB265610 (CXCR2 inhibitor, #SML0421) was purchased from SIGMA; Primary antibodies: NF-κB P65(C22B4) Rabbit mAb (#4764S), Phospho-IKKα/β (Ser176/180) Rabbit mAb (#2697), p44/42 MAPK (Erk1/2) Rabbit mAb (#4695) , Phospho-p44/42 ( Thr202/Tyr204) Antibody (#9101) , Phospho-Stat3 (Tyr705) Rabbit mAb (#9145) purchased from CST; Rabbit Anti-CXCR2 antibody (#ab14935), Rabbit Anti-CXCL5 antibody(#ab14935), Rabbit Anti-CXCR2 antibody(#ab14935), Rabbit Anti-CXCL5 antibody(#ab9983) were purchased from Abcam; secondary antibody: GAPDH Mouse monoclonal antibody(#MAB3740) was purchased from MILLIPORE.

### TCGA Data Acquisition

For this study, we utilized the TCGA (The Cancer Genome Atlas) data repository (https://tcga-data.nci.nih.gov/tcga/) as the primary source of data. We downloaded transcriptome analysis data (HTSeq-Counts and HTSeq-FPKM) from the TCGA-COAD project using the R (version R 4.1.0) package called TCGAbiolinks. The HTSeq-FPKM data underwent a log2(FPKM+1) transformation, while HTSeq-Counts data was used for conducting differential analysis.

### Differentially Expressed Genes

To identify differentially expressed genes (DEGs), we analyzed the expression levels of S100A8 and S100A9 (HTSeq-Counts) with the R package DESeq2. We set thresholds of |log2FoldChange| > 1 and an adjusted P-value < 0.05. Venn diagrams were generated using an online tool (http://bioinformatics.psb.ugent.be/webtools/Venn/) to visualize the crossover genes among the DEGs.

### Gene Set Enrichment Analysis

To gain insights into the functions of the DEGs, we performed gene ontology (GO) analyses, including biological process (BP), cellular component (CC), and molecular function (MF), using the "clusterprofiler" R package. The results were plotted using the "ggplot2" R package. Additionally, we conducted Gene Set Enrichment Analysis (GSEA) using the R package "clusterProfiler" to identify significant functional differences associated with S100A8 and S100A9 expression. We considered results with a standardized enrichment score (|NES| > 1), a p-value < 0.05, and an FDR (false discovery rate) q-value < 0.05 as statistically significant.

### Protein-Protein Interaction Network Construction and Central Gene Identification

To explore the interactions among intersecting genes, we constructed protein-protein interaction (PPI) networks using the STRING database (https://string-db.org/). The DEGs that intersected with the PPI networks were imported into Cytoscape v3.9.1, and hub genes were identified using the BottleNeck algorithm in Cytohubba.

### Reverse Transcription and Real-time Quantitative PCR

To extract total RNA from cells and tissues, we employed Trizol (Invitrogen, CA). Subsequently, cDNA synthesis was performed using 2 μg of total RNA and the Revert Aid First Strand cDNA Synthesis Kit (Thermo, MD). The primers used for quantitative real-time PCR (qRT-PCR) can be found in Supplementary Table 1. Relative mRNA levels of the target genes were determined as the ratio of the target gene to GAPDH and were calculated using standard curves. The reported results represent the average ratios from three independent experiments.

### Inflammatory Factor Protein Microarray Experiments

For the inflammatory factor protein microarray experiments, we transfected mouse colon cancer cells CT26.WT with expression plasmids containing pIRES-mS100A8, pIRES-mS100A9, or both. After 24 hours, we collected the cell culture supernatants for protein microarray assay using the Mouse Inflammation Array G1 (4)4 Sample Kit (#AAM-INF-G1-4) from RayBiotech.

### Western Blotting

Cell harvesting was performed using lysis buffer (Roche, IN) supplemented with phosphatase and protease inhibitors. We then separated aliquots of 50 μg of total protein using SDS-PAGE (Bio-Rad, CA) and transferred them onto polyvinylidene difluoride membranes (Millipore, MA, USA). The membranes were probed with primary antibodies overnight at 4°C, followed by incubation with secondary antibodies for 1 hour at 37°C. The signal detection was carried out using the Chemidoc XRS+ system (Bio-Rad).

### Enzyme-Linked Immunosorbent Assay (ELISA)

Mouse colon cancer cells CT26.WT were transfected with expression plasmids containing pIRES-mS100A8, pIRES-mS100A9, or both constructs together, respectively. After 24 hours, cell culture supernatants were collected for analysis. The levels of inflammatory factors were quantified using an enzyme-linked immunosorbent assay (ELISA) kit obtained from USCN (Wuhan, China).

### Immunohistochemistry

Paraffin-embedded sections, with a thickness of 4 μm, were prepared and subsequently deparaffinized and rehydrated. Immunohistochemical staining was performed according to previously established protocols to assess the expression of S100A8, S100A9, p-Akt1, p-Smad5, Id3, and p21. Slides with immunostained samples were observed under a microscope, and the scoring was done based on the percentage of positive cells. The scoring system ranged from 0 to 4 as follows: 0 for no staining, 1 for less than 10% positive cells, 2 for 11-50% positive cells, 3 for 51-75% positive cells, and 4 for more than 75% positive cells. The evaluation of staining was performed in a blinded manner by at least two pathologists. Samples with expression scores greater than or equal to 2 were classified as having high expression, while those with scores less than 2 were considered to have no or low expression.

### Cell Invasion Assay

To assess cell invasion, an 8 μm Transwell chamber (Corning, NY, USA) with a Matrigel basement membrane was used. CT26.WT, SW480, and SW620 cells were transfected with the respective constructs and suspended in 200 µl of serum-free medium. The cells were then seeded onto the Matrigel-coated inserts of a 24-well culture plate, while the lower chamber was filled with normal growth medium. After seeding, the cells were allowed to migrate for 24, 48, and 72 hours. Non-invasive cells on the upper side of the chamber were removed by scrubbing, while invasive cells on the lower surface of the membrane were fixed in methanol, sealed, and dried. Technicians, unaware of the experimental conditions, counted the number of invading cells in four randomly selected microscopic fields of view for each filter. The assay was independently repeated three times to ensure accuracy and reproducibility.

### MTT Assay

The MTT assay was conducted using 3-(4,5-dimethyl-2-yl)-2,5-diphenyl-2II-tetrazolium bromide (MTT) purchased from Sigma (Missouri, USA). CT26.WT cells transfected with the plasmid were seeded in 96-well plates at a density of 3000 cells per well. At 24, 48, 72, and 96 hours post-transfection, 20 μl of MTT solution (5 mg/ml) was added to the corresponding treatment groups. The cells were then incubated for an additional 4 hours at 37°C under light-avoidance conditions. The resulting formazan crystals were dissolved, and the optical density (OD) was measured at 490 nm to assess cell proliferation.

### Tumour Formation in Nude Mice

For evaluating the impact of S100A8 on colon cancer cell proliferation and lung metastasis, five-week-old male thymus-less BALB/c nude mice were used. CT26.WT cells (4 × 10^6) transfected with S100A8 or an empty control expression vector were injected into the tail vein of the mice. After 35 days, necropsy was performed, and photographs of the entire lung tissue were taken. The number of metastases on the surface of the five lung lobes was counted under a dissecting microscope. Immunohistochemistry was used to analyze tumour cells in lung metastases from individual mice. The experiments were conducted with five mice per group, and all animal procedures were performed following institutional guidelines.

### Subcutaneous Tumour Formation Experiments in Nude Mice

Subcutaneous tumour formation was assessed using the prepared CT26.WT cells as described above. The cell concentration was adjusted to 1 × 10^7/ml. A small portion of the cells was retained for detecting S100A8 transfection efficiency. Subcutaneous injections were made into the right armpit of the nude mice. The mice were observed for 10 minutes after injection before being placed back into their rearing cages for further observation of their activities and tumour size measurement every 3 days. After 15 days, the mice were euthanized, and the subcutaneous tumours were removed and weighed.

### Statistical Analysis

Data analysis was performed using SPSS 17.0 and GraphPad Prism 5, and R 4.1.0 was used for data visualization. Categorical variables, such as histological type of colorectal tumour, age, sex, metastasis, and degree of differentiation, were assessed using the χ^2 test or Kruskal-Wallis H test. Independent t-tests or ANOVA were performed for qRT-PCR and MTT analyses. The significance level was set at * p < 0.05.

## Results

### Increased Expression of S100A8 and S100A9 in Tissues from Colon Cancer Patients

**Figure 1:**
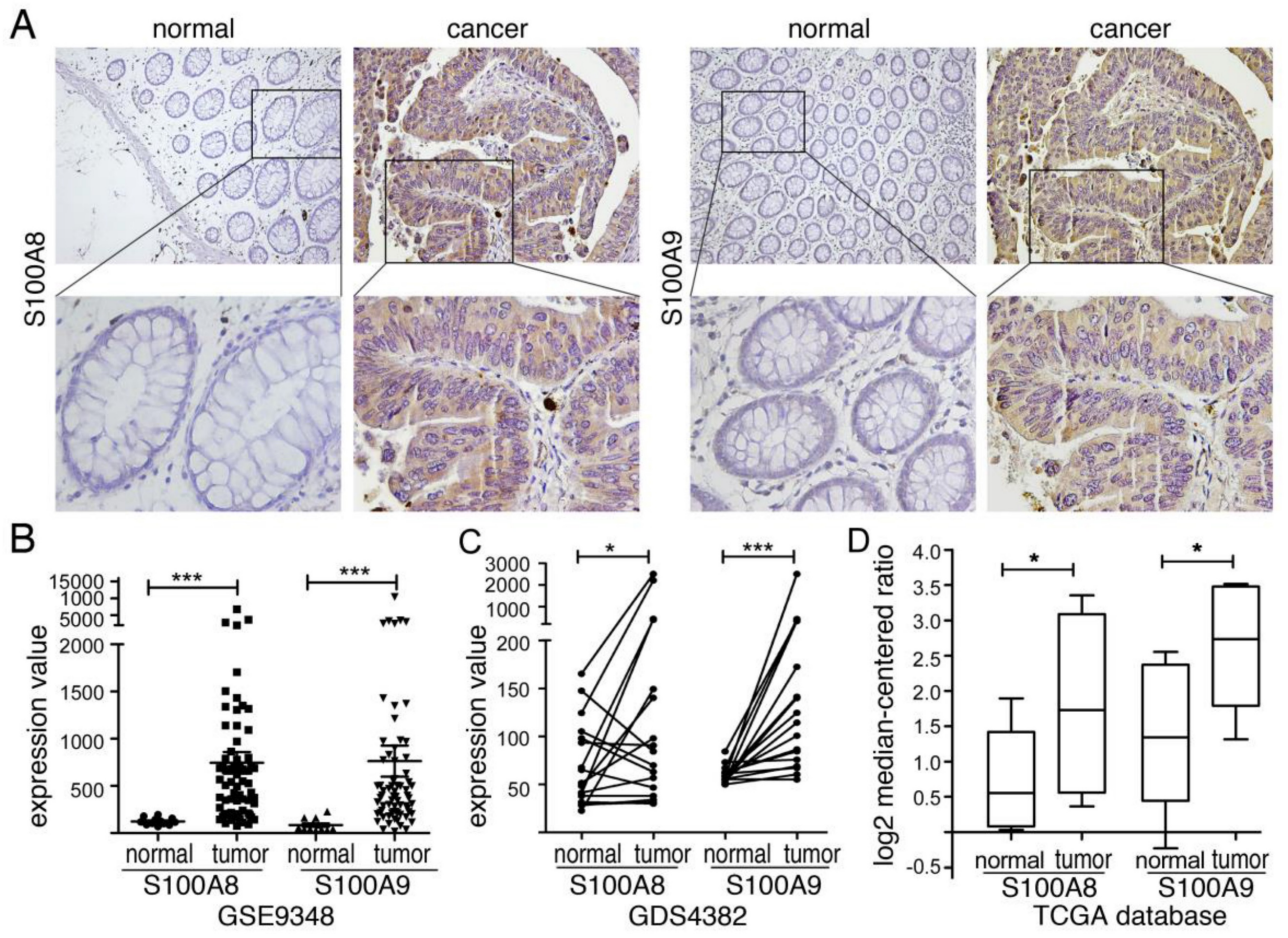
S100A8 and S100A9 are expressed in tumor tissues of colon cancer patients. (A) Immunohistochemical analysis was conducted to detect the expression of S100A8 (left) and S100A9 (right) in human colon cancer tissues. Positive reactions were indicated by a brown color, demonstrating intense staining in the plasma of tumor cells. The images were captured at both low magnification (100×) and high magnification (400×); (B) Gene expression profiling microarray analysis from the GEO database (GSE9348) was performed to assess the expression of S100A8 and S100A9. The analysis included 12 cases of normal intestinal mucosa (normal) and 70 cases of colorectal cancer (tumor). (C) Another gene expression profiling microarray analysis from the GEO database (GDS4382) focused on 17 pairs of colon cancer tissues (tumor) and their corresponding paracancerous tissues (normal) to examine the expression of S100A8 and S100A9. (D) The TCGA database was utilized for the analysis of S100A8 and S100A9 expression. This analysis involved 22 colon cancer tissues (tumor) and 22 samples of normal intestinal mucosa (normal). Statistical analysis was performed using the Student's t-test, with significance levels indicated as *P < 0.05, **P < 0.01, and ***P < 0.001.

S100A8/A9, a protein complex, is primarily found in inflammatory cells that infiltrate colorectal cancer tissues 29. Our previous findings indicated a significant increase in both RNA and protein levels of S100A8 and S100A9 in colorectal cancer tissues, suggesting a correlation with the malignancy and prognosis of the lesions 30. To further investigate their expression in CRC, we utilized data from the GEO and TCGA databases. Our analysis revealed significant upregulation of S100A8 and S100A9 in colon cancer tissues compared to normal intestinal mucosa in the GSE9438 dataset (Figure 1B). Similarly, the GDS4382 dataset showed significantly higher expression of S100A8 and S100A9 in cancer tissues compared to corresponding paracancerous tissues (Figure 1C) (P<0.05). Analysis of TCGA data yielded consistent results (Figure 1D). Furthermore, our Western blot analysis revealed heightened expression levels of S100A8 and S100A9 in colorectal cancer cells SW480, SW620, and CT26 when compared to normal colonic epithelial cells NCM460 (Supplementary Figure 1). To better understand the localization of S100A8 and S100A9 in cancer tissues, we conducted immunohistochemical analyses on human colorectal cancer samples. The results not only confirmed increased expression of S100A8/A9 in mesenchymal cells but also showed a significant elevation in tumor glandular epithelial cell plasma compared to normal tissues (Figure 1A).

### S100A8 and S100A9 are Associated with the Expression of Inflammatory Factors in Colorectal Cancer

**Figure 2:**
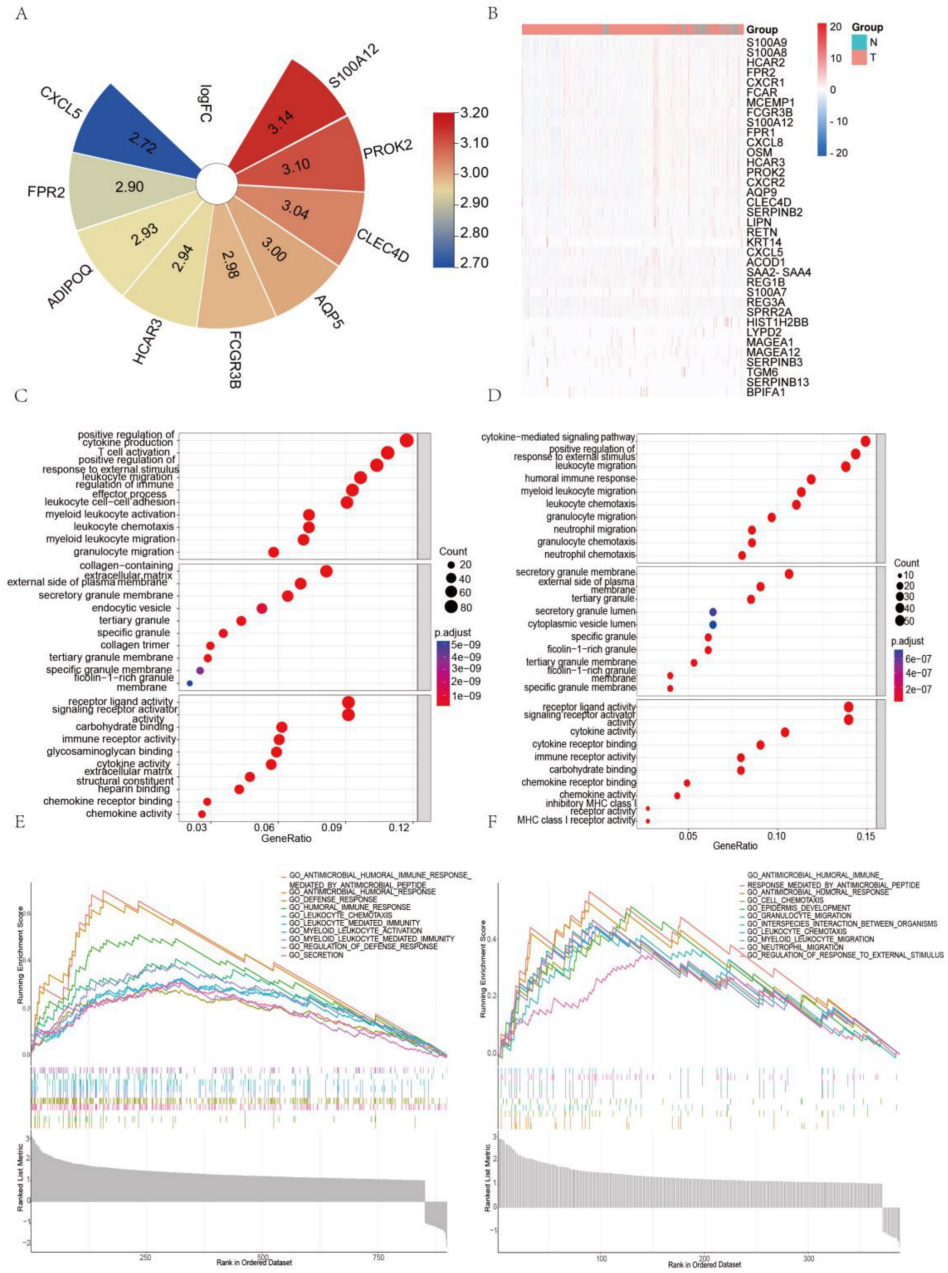
Correlation of S100A8 and S100A9 with the expression of inflammatory factors in colorectal cancer. (A) Heatmap showing up-regulated genes identified after differential analysis of S100A8. (B) Heatmap depicting up-regulated genes identified after differential analysis of S100A9. (C) GO enrichment analysis results for the differential genes associated with S100A8. (D) GO enrichment analysis results for the differential genes associated with S100A9. (E) GSEA pathway enrichment analysis results for the differential genes of S100A8. (F) GSEA pathway enrichment analysis results for the differential genes of S100A9."

We obtained the expression matrix for colorectal cancer patients from the TCGA database and conducted a differential analysis based on the expression levels of S100A8 and S100A9. We identified genes with a significant P-value <0.05 and a fold change (logFC) greater than 1. These are presented in the heatmap (Figure 2A-B) and were further subjected to GO and GSEA enrichment analyses. Figure 2C-D displays the top 10 significantly enriched biological processes, cellular components, and molecular functions according to GO terms, with a particular focus on DEGs that contribute to cytokine production. Additionally, we examined the top ten significantly enriched functional pathways using GSEA (Figure 2E-F). Our analysis highlighted several key pathways related to inflammation within the MSigDB enrichment set (c5.cp.v7.0.symbols.gmt), including humoral immune response, modulation of the defense response, chemotaxis of leukocytes, myeloid leukocyte migration, and neutrophil migration.

### S100A8 and S100A9 Overexpression Induces Secretion of Inflammatory Factors in Colon Cancer

**Figure 3:**
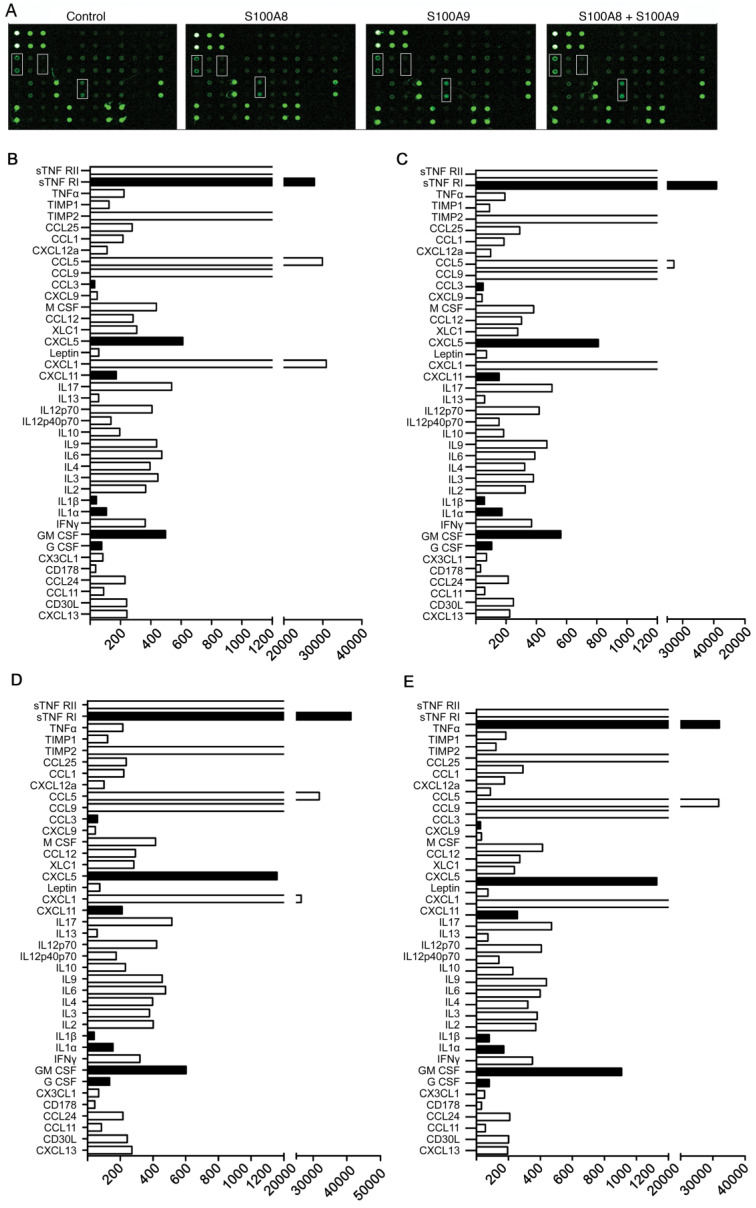

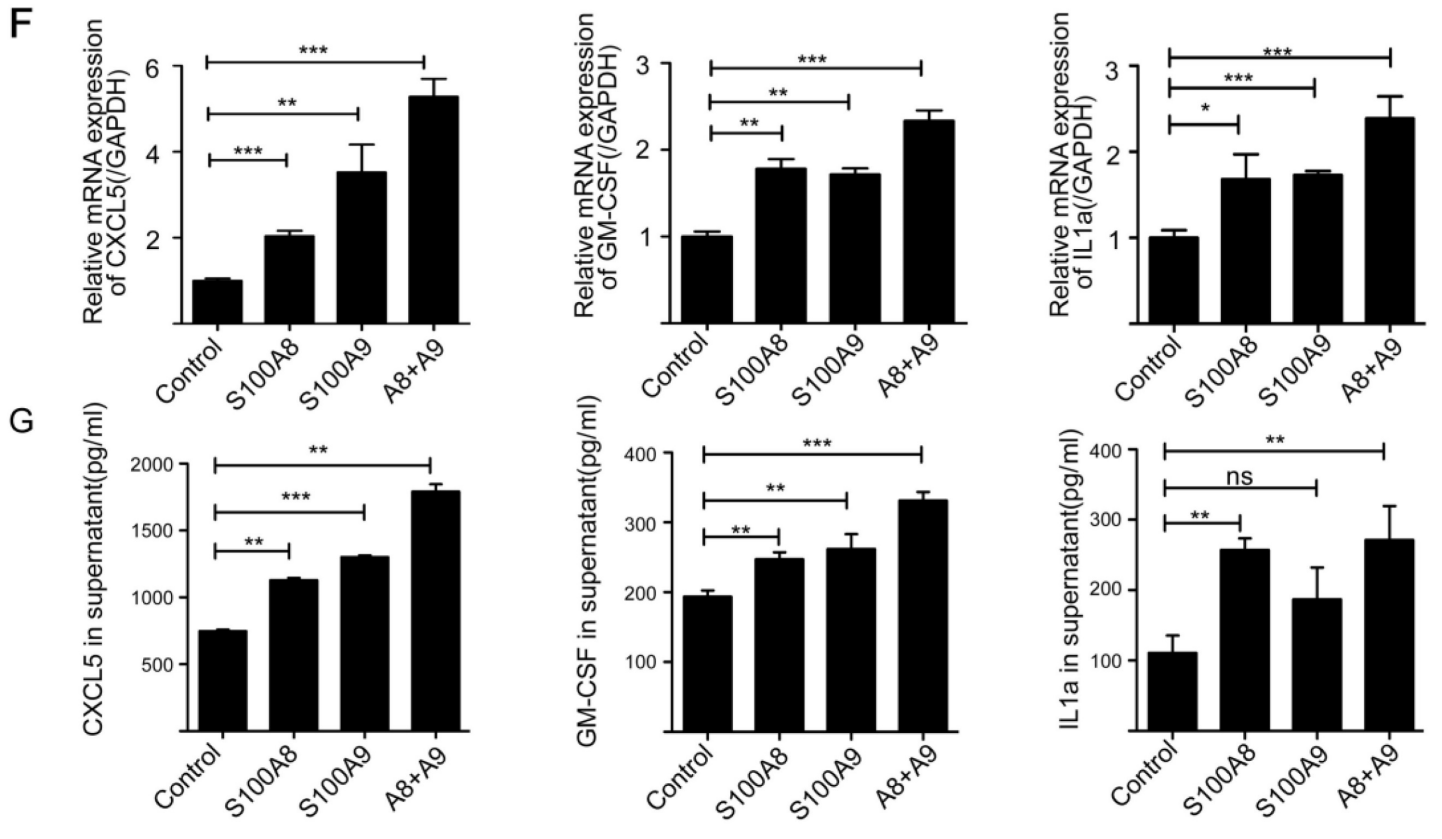
Induction of inflammatory factor secretion in colon cancer cells by S100A8 and S100A9. (A) Protein microarray analysis revealing the expression levels of inflammatory factors in cell culture supernatants. CT26 cells were transfected with S100A8, S100A9, or both, with white boxes indicating CXCL5, GM-CSF, and IL1a, respectively. Quantitative results of the microarray scan data for the NC group (B), S100A8 group (C), S100A9 group (D), and S100A8/A9 co-transfected group (E). Black bands represent significantly elevated levels of inflammatory factors, including sTNF RI, CCL3, CXCL5, CXCL11, IL1a, IL1b, GM-CSF, and G-CSF. (F) Real-Time PCR analysis demonstrating altered expression of CXCL5, GM-CSF, and IL1a. (G) ELISA validation of the increased expression of CXCL5, GM-CSF, and IL1a in cell supernatants. Data were obtained as mean±SD from three replicate experiments, and Statistical analysis was performed using the Student's t-test, with significance levels indicated as *P < 0.05, **P < 0.01, ***P < 0.001., and ns indicates no significance.

To investigate the impact of elevated S100A8 and S100A9 expression on the secretion of inflammatory factors by colon cancer cells, we transfected CT26 colon cancer cells with overexpression plasmids containing S100A8 and mS100A9, both individually and in combination. Subsequently, cell supernatants were collected for protein microarray assays. The results revealed that overexpression of S100A8/S100A9 led to varying levels of increased secretion of cytokines, including CXCL5, CXCL11, GM-CSF, G-CSF, IL1a, IL1b, sTNF RI, and CCL3 (Figure 3 A-E). To validate these findings, we used ELISA to assess the expression of CXCL5, GM-CSF, IL1a, and other proteins in the cell culture supernatants obtained through the aforementioned experimental procedure. The results were consistent with the protein microarray data (Figure 3G). Additionally, we collected RNA from the same cells and further confirmed the observations using real-time PCR analysis, which demonstrated a significant increase in mRNA expression levels of CXCL5, GM-CSF, and IL1a following transfection with S100A8 and S100A9 expression plasmids (Figure 3F). In conclusion, our findings strongly suggest that S100A8/A9 promotes the expression and secretion of a diverse range of inflammatory factors.

### S100A8 and S100A9 Induce CXCL5, GM-CSF, and IL1a Secretion via the Inflammatory Signaling Pathway

**Figure 4:**
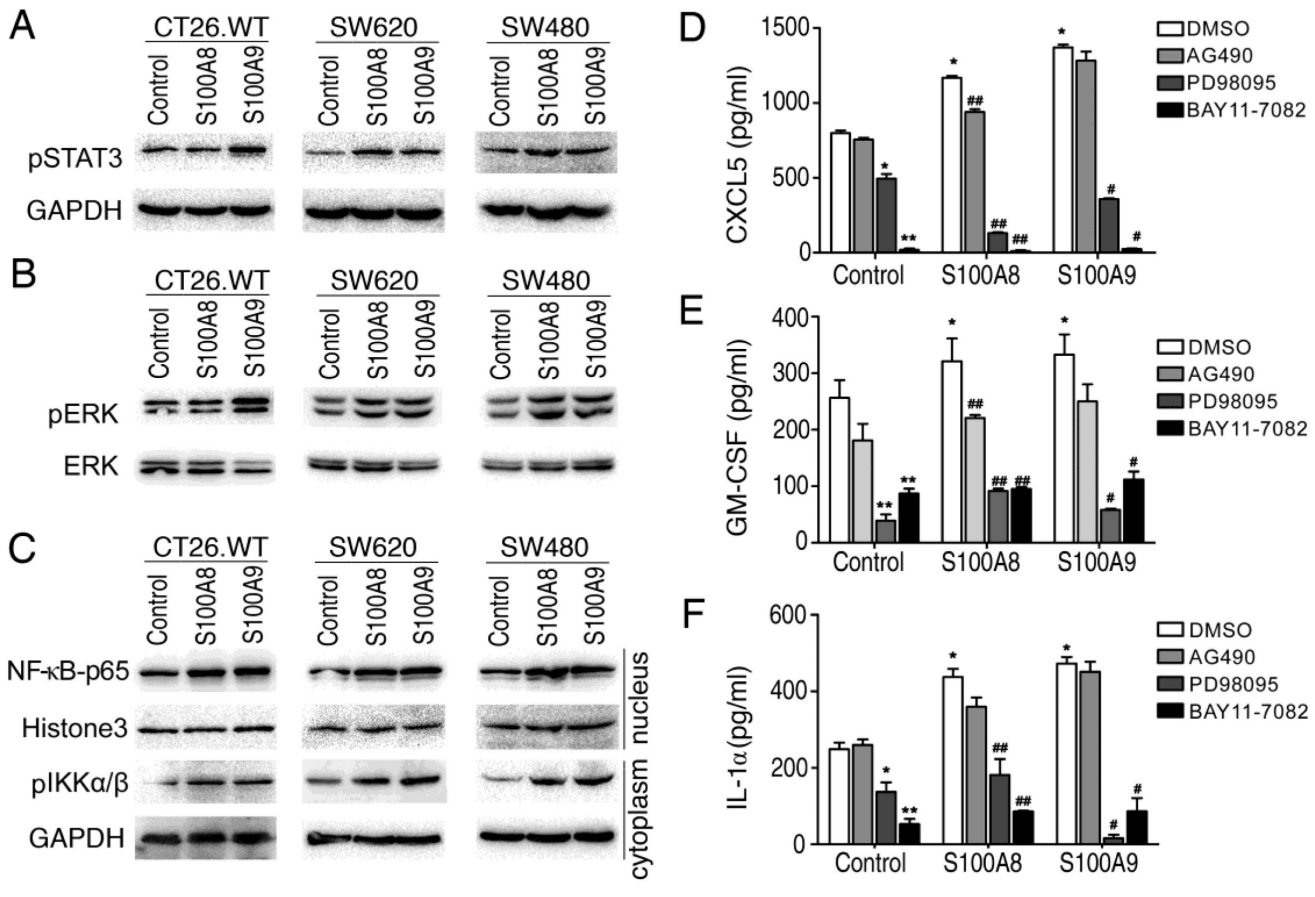
S100A8 and S100A9 activate STAT3, ERK-MAPK and NF-κB signaling pathways and regulate the secretion of inflammatory factors. The CT26, SW620, and SW480 cell lines were transfected with S100A8 or S100A9 expression vectors and cultured conventionally for 24 hours. (A) Western blotting was performed to detect the levels of phosphorylated STAT3. (B) The levels of phosphorylated ERK were examined. (C) The entry of NF-kB p65 into the nucleus and the levels of phosphorylated IKKα/β were investigated. Additionally, CT26 cells overexpressing S100A8 and S100A9 were treated with inhibitors AG490, PD098059, and BAY11-7082. The levels of CXCL5 (D), GM-CSF (E), and IL1a (F) in the cell supernatant were measured using ELISA. Comparisons with the Control+DMSO group are denoted as * (P < 0.05), ** (P < 0.01), and *** (P < 0.001). Comparisons with the respective DMSO groups are denoted as # (P < 0.05) and ## (P < 0.01).

To understand the potential mechanisms underlying the expression of inflammatory factors induced by intracellular S100A8 and S100A9, we explored their impact on signaling pathways associated with inflammation. Utilizing Western blotting, we examined three colon cancer cell lines (CT26, SW620, and SW480) that were genetically modified to overexpress S100A8 and S100A9. The results showed that intracellular overexpression of S100A8 or S100A9 significantly increased the phosphorylation of STAT3 (Figure 4A) and ERK (Figure 4B). Additionally, we observed an elevation in IKKα/β expression, accompanied by an increase in the nuclear entry of NF-κB P65 (Figure 4C). These findings suggest that intracellular S100A8 and S100A9 can activate the NF-κB, ERK-MAPK, and STAT3 signaling pathways.

To further assess whether the induction of inflammatory factor synthesis and secretion by S100A8 and S100A9 depends on the activation of these signaling pathways, we treated the supernatants of CT26 cells transfected with S100A8 or S100A9 with inhibitors targeting these pathways and conducted an ELISA. The levels of CXCL5, GM-CSF, and IL1a in the supernatant decreased when treated with the JAK/STAT3 pathway inhibitor (AG490), although not significantly. However, when treated with the ERK inhibitor (PD98059) and NF-κB inhibitor (BAY11-7082), the levels of CXCL5, GM-CSF, and IL1a were significantly diminished or even undetectable (Figure 4D-F). This indicates that the activation of NF-κB and ERK-MAPK signaling pathways plays a crucial role in the induction of inflammatory factors by intracellular overexpression of S100A8/S100A9, while the JAK/STAT3 pathway may have only partial involvement.

### S100A8 Promotes Proliferation and Invasion of Colon Cancer Cells Through the CXCL5/CXCR2 Axis

**Figure 5:**
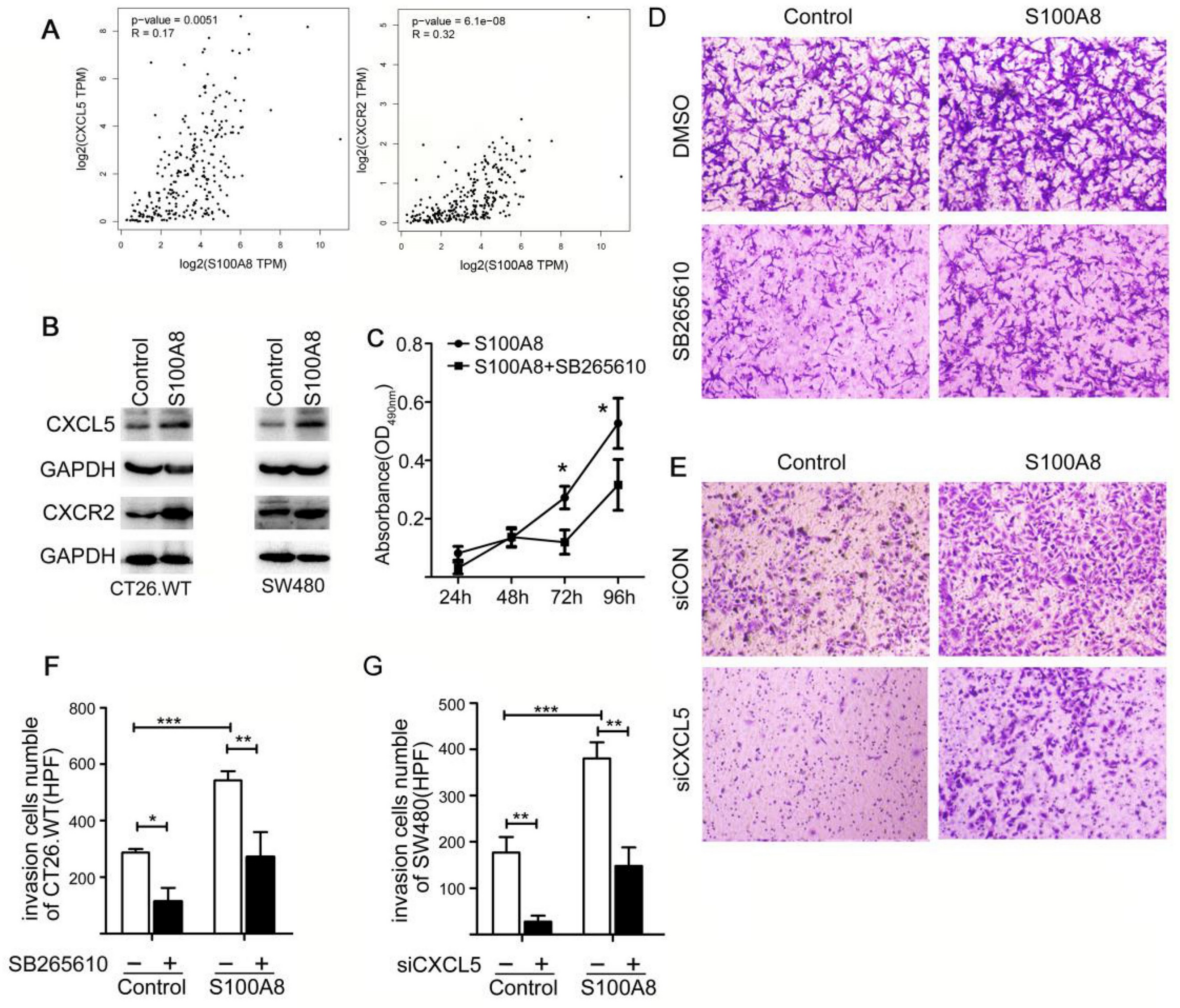
S100A8 Induce Proliferation and Invasion in Colon Cancer Cells via CXCL5/CXCR2 Pathway. (A) Analysis of Correlation between S100A8 and CXCL5/CXCR2. (B) Western blot analysis of CXCL5 and CXCR2 expression 24 hours post transfection with S100A8 overexpression plasmid in CT26 or SW480 cell lines. (C) Post S100A8 transfection, cells were treated with SB265610, and MTT absorbance values were measured at 24h, 48h, 72h, and 96h; DMSO treatment served as the control. (D) CT26 cells, transfected with S100A8, were subjected to treatment with 1uM SB265610 or an equal amount of DMSO as control. (E) SW480 cells overexpressing S100A8 were concurrently transfected with siCXCL5; siCON was employed as the control. (F) Enumeration of cells traversing 5 high-magnification fields after transfection with S100A8 and subsequent treatment with SB265610. (G) Quantification of cells crossing 5 high-magnification fields following overexpression of S100A8 in SW480 cells and siCXCL5 transfection. Statistical analysis was performed using the Student's t-test, with significance levels indicated as *P < 0.05, **P < 0.01, and ***P < 0.001.

Previous studies have confirmed the pivotal role of the extracellular secretory proteins S100A8/S100A9 in promoting the proliferation of tumor cells and facilitating invasive metastasis. These proteins mainly exert their influence by engaging with receptors such as RAGE and TLR4. We discovered that the expression of S100A8 in colon cancer cells led to increased levels of CXCL5 and its corresponding receptor, CXCR2 (Figure 5B). Subsequently, we explored the relationship between S100A8 expression and CXCL5/CXCR2 in patients with CRC using TCGA database, revealing a robust positive correlation (Figure 5A). This led us to further investigate the potential impact of elevated intracellular S100A8 concentrations on the malignant behaviors of tumor cells and whether this mechanism involves inflammatory mediators and their corresponding receptors.

To assess the influence of S100A8 on tumor cell behavior, we conducted MTT and Transwell invasion assays, both performed 24 hours post-transfection of CT26 cells with an S100A8 overexpression plasmid. Notably, the introduction of the CXCR2 inhibitor (SB265610) into the cell culture medium notably mitigated the proliferative impact of S100A8 (Figure 5C, D). Moreover, Transwell invasion assays showed that S100A8 transfection substantially increased the invasiveness of colon cancer cells, facilitating their penetration through the matrix gel and migration to the lower compartment. The treatment of cells with SB265610 significantly inhibited this enhanced cellular invasion (Figure 5C, F), with all observed variances indicating statistical significance. To validate these findings, we repeated the invasion experiments using human-derived colon cancer cells SW480, yielding consistent results (Figure 5E, G). In conclusion, our findings offer compelling evidence that S100A8 amplifies the proliferative and invasive properties of colon cancer cells by activating the CXCL5/CXCR2 pathway. This underscores the critical role of inflammatory factors and their associated receptors in promoting tumor malignancy.

### S100A8 Overexpression Enhances Tumorigenesis and Metastasis of Colon Cancer Cells via CXCR2

**Figure 6:**
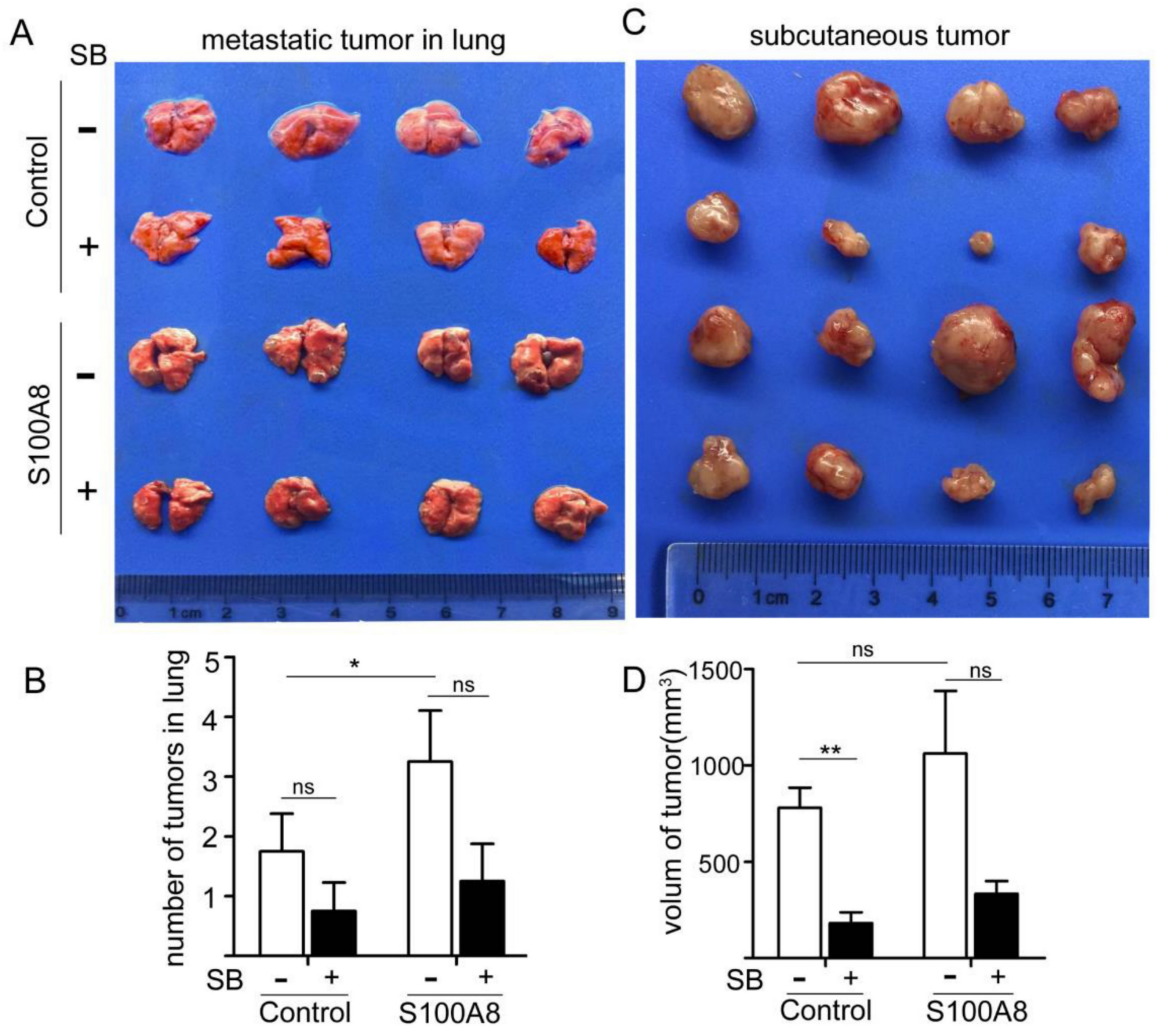
In Vivo Experiments Confirm the Role of S100A8 in Promoting Colon Cancer Cell Proliferation and Metastasis via CXCR2. To further validate the impact of S100A8 in vivo, CT26 cells were transfected with S100A8 for 24 hours, followed by tail vein or left axillary subcutaneous injection in mice. Intraperitoneal treatment with the CXCR2 inhibitor SB265610 was administered. (A) and (B) depict the status and number of metastatic tumors in the lung upon sacrifice after 30 days of treatment, respectively. (C) and (D) present the status and volume of axillary transplanted tumors assessed after 15 days of treatment (volume = length diameter × width diameter 2/2). Statistical analysis was performed using the Student's t-test, with significance levels indicated as *P < 0.05, **P < 0.01, and ns indicating no significance. SB denotes SB265610.

To validate our findings, we established both subcutaneous and tail vein tumor models in nude mice using CT26 cells that overexpressed S100A8. In the tail vein model, cells were injected into the tail vein, and starting from day 2, the CXCR2 inhibitor, SB265610 (1 mg/kg-d), was administered intraperitoneally every other day. Starting on day 8, the dose was increased to 2 mg/kg-d. The control group received an equivalent concentration of DMSO. On day 30, the mice were sacrificed, and their lungs were isolated for examination (Figure 6A). The number of lung metastases was also quantified (Figure 6B). We observed that S100A8-transfected colon cancer cells formed significantly more lung metastases than did cells transfected with the empty vector (P<0.01). However, the number of metastases decreased following SB265610 treatment, although the difference was not statistically significant.

In the subcutaneous tumor transplantation experiment with nude mice, the same methodology was employed, and measurements were taken after 15 days (Figure 6C). The volumes of the transplanted tumors were calculated (Figure 6D). We found that the volume of tumors in the axillary transplantation site of the S100A8-transfected group was larger than that in the empty vector group (1061.4±651.3 mm3 vs. 778.8±211.1 mm3). Administration of SB265610 led to a reduction in tumor volume. These observations suggest that intracellular S100A8 activates CXCR2, thereby accelerating cancer progression and metastasis in vivo.

## Discussion

Epidemiological and molecular investigations have illuminated the close correlation between intestinal inflammation and both the onset and progression of colorectal cancer 31-33. In a prior study, we established a dynamic mouse model that simulates the transition from inflammation to dysplasia and eventually to colorectal cancer 8. In this model, we observed a marked increase—in some instances, dozens to hundreds of times—in the levels of calcium-binding proteins, specifically S100A8 and S100A9, at every pathological stage compared to the control group 30. This heightened expression consistently coincided with the persistent activation of key signaling pathways, including NF-κB, STAT3, MAPK, and Wnt/β-catenin. These findings strongly suggest that the inflammatory cytokines S100A8 and S100A9, as well as associated signaling pathways and their downstream target genes, play pivotal roles in the transition from inflammation to carcinoma. Using diverse techniques like ELISA, immunohistochemistry, and biodatabase analysis, we found that S100A8 and S100A9 expression levels were significantly elevated in colorectal cancer patient tumor tissues compared to normal tissues. Furthermore, differential analysis and pathway enrichment studies revealed a strong correlation between the levels of S100A8/S100A9 and inflammatory signaling pathways in these patients, underscoring their essential roles in the advancement and progression of colorectal cancer.

S100A8 and S100A9 are primarily expressed in various inflammatory cells and constitute a significant portion of the cellular weight in neutrophils and monocytes 34. Known as calreticulins, these proteins form heterodimers with antimicrobial effects 22. Research has emphasized the utility of fecal calprotectin as a biomarker for evaluating the progression of intestinal inflammatory diseases such as IBD 35-38. Notably, S100A8 and S100A9 were not confined to increased expression solely in mesenchymal immune cells; their expression was also notably elevated within tumor cells, exceeding levels found in normal tissues. This phenomenon has been observed in various cancers, including gastric, liver, lung, prostate, and breast cancers 39-42. Our immunohistochemical analyses further confirmed the significant increase in the cytoplasmic presence of S100A8 and S100A9 in the glandular epithelial cells of colorectal cancer tumors compared to normal tissues.

Cellular signaling pathways serve as vital conduits for executing biological functions and act as crucial upstream triggers for the synthesis and release of inflammatory factors 43. They are frequently involved in tumorigenesis 44. Transfection of S100A8 or S100A9 expression vectors into colon cancer cells significantly activated NF-κB, STAT3, and ERK-MAPK, highlighting the role of S100A8/A9 as potent cancer promoters by orchestrating key connections within the signaling cascade. Our studies employed inhibitor experiments to elucidate the regulation of these cytokines through these signaling pathways, with a particular focus on the nearly complete suppression of inflammatory mediators such as CXCL5, GM-CSF, and IL1a following the inhibition of NF-κB p65 and ERK-MAPK pathways. This laid the groundwork for constructing a positive feedback signaling transduction pathway involving "inflammatory factor-S100A8/A9-signaling pathway-inflammatory factor."

Our protein microarray analyses revealed significant alterations in the expression of CXCL5, with the CXCL5/CXCR2 axis playing a pivotal role in driving tumor cell deterioration, as documented in previous studies 45, 46. Recombinant CXCL5 was found to stimulate tumor cell proliferation and invasive metastasis, an effect abrogated by CXCR2-neutralizing antibodies 47. Notably, S100A8 significantly promotes colon cancer cell proliferation and invasive metastasis. We investigated whether phenotypic changes induced by S100A8 could be counteracted or inhibited by employing a CXCR2 inhibitor (SB265610) or an siRNA targeting CXCL5 to disrupt the CXCL5/CXCR2 signaling axis. SB265610, a small-molecule inhibitor, competes for binding to the CXCR2 receptor, thereby blocking the biological effects induced by endogenous ligands like CXCL5^48^, and shows antitumor activity. Inhibitor experiments confirmed our hypothesis, validating that S100A8 enhances the proliferation of colon cancer cells and boosts their invasive metastatic potential through the CXCL5/CXCR2 bioaxis pathway.

In conclusion, our study effectively illustrates the heightened expression of S100A8/S100A9 within colorectal tumor epithelial cells, indicating their crucial role in tumor advancement. These proteins act as pivotal drivers, instigating the expression of various inflammatory factors through the activation of NF-κB and ERK-MAPK signaling pathways. Particularly noteworthy is S100A8's involvement in establishing a self-reinforcing feedback loop typified by 'inflammation-tumor-inflammation' within colorectal cancer cells.

However, while our study sheds light on the significance of S100A8 and S100A9 in colorectal cancer progression, it's imperative to acknowledge the possibility of heightened S100A8 expression being secreted through the cell membrane into the tissue interstitial space, thereby influencing the process via an exogenous pathway. This raises inquiries regarding their extracellular effects and potential impact on neighboring cells. Additionally, although we centered our investigation on the CXCL5/CXCR2 axis as a primary signaling pathway, it's crucial to recognize that other signaling cascades may also contribute to colorectal cancer progression. Moreover, our study's reliance on in vitro experiments and a limited selection of cell lines may not fully encapsulate the complexity of the tumor microenvironment in vivo. These considerations underscore the necessity for future studies to broaden the scope of our findings and facilitate a deeper understanding of the intricate interactions between S100A8, S100A9, and the tumor microenvironment in colorectal cancer.

## Supplementary Material

Supplementary figure.
