# Supplementary figures and images for: S100A8-Mediated Inflammatory Signaling Drives Colorectal Cancer Progression via the CXCL5/CXCR2 Axis
Source: J Cancer. 2024 Apr 29;15(11):3452–65. doi: 10.7150/jca.92588 (PMC11134430; doi:10.7150/jca.92588)

Supplementary Figure 1

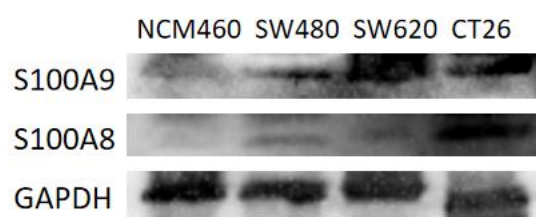

Supplement: Supplementary file 1 — Supplementary figure. [file jcav15p3452s1.pdf]
